# Genetic associations in sepsis and ARDS

**DOI:** 10.3389/fphar.2026.1864880

**Published:** 2026-08-24

**Authors:** Jonathan J. Moroniti, Poorna Manappurathu Rameshchandran, Mengqi Guo, Victoria Labuda, Mark Chandy, Aleksandra Leligdowicz

**Affiliations:** 1 Department of Medicine, Schulich School of Medicine and Dentistry, Western University, London, ON, Canada; 2 Robarts Research Institute, Schulich School of Medicine and Dentistry, Western University, London, ON, Canada; 3 Department of Microbiology and Immunology, Schulich School of Medicine and Dentistry, Western University, London, ON, Canada; 4 Lawson Health Research Institute, London Health Sciences Centre, London, ON, Canada; 5 Department of Physiology and Pharmacology, Schulich School of Medicine and Dentistry, Western University, London, ON, Canada

**Keywords:** ARDS, critical illness, genetics, immune regulation, sepsis, SNPs, vascular injury

## Abstract

Critical illness syndromes, such as sepsis and acute respiratory distress syndrome (ARDS), are characterized by substantial clinical heterogeneity and remain major causes of morbidity and mortality worldwide. Increasing evidence suggests that genetic variation contributes to susceptibility, disease severity, and clinical outcomes in critically ill patients. However, the molecular mechanisms linking genetic predisposition to the pathophysiology of sepsis and ARDS remain incompletely understood. In this review, we evaluated genetic associations reported in sepsis and ARDS, including 13 genome-wide studies identifying 19 unique single-nucleotide polymorphisms (SNPs) across 17 distinct genomic loci, as well as 21 meta-analyses of candidate-gene studies identifying 21 SNPs across 16 genes. The identified variants were primarily associated with pathways involved in pathogen recognition, immune and inflammatory signaling, leukocyte recruitment, and endothelial dysfunction. Collectively, these findings support a polygenic basis for susceptibility to critical illness and highlight several biologically relevant pathways that may contribute to sepsis and ARDS pathogenesis. Improved understanding of the functional consequences of these variants may facilitate the identification of potential therapeutic targets and support the development of precision-guided approaches to critical care.

## 1 Introduction

Sepsis and acute respiratory distress syndrome (ARDS) are complex critical illness syndromes characterized by a combination of signs and symptoms, with non-specific diagnostic criteria (Maslove et al., 2022; Singer et al., 2016). Critically ill patients differ in age, comorbidities, anatomic source of infection, infecting organisms, and genetic predisposition (Arina and Singer, 2021). Many genetic polymorphisms, including single-nucleotide polymorphisms (SNPs), have been associated with increased susceptibility to sepsis and ARDS, as well as poorer clinical outcomes (Boyd et al., 2014; Sutherland and Walley, 2009). However, the molecular mechanisms of how genetic polymorphisms contribute to the pathophysiology of critical illness remain unclear.

Genetic association studies in critical illness have evolved over the past 3 decades. Early studies adopted a candidate gene approach, in which pre-selected, hypothesis-driven genes were selected due to their association with immune responses (Sutherland and Walley, 2009). A substantial proportion of these studies produced conflicting results, with many reporting divergent effects for the same SNP. For instance, multiple investigations of Toll-like receptor 4 (*TLR4*) variants reported inconsistent associations with sepsis risk, and a meta-analysis of 17 studies ultimately found no overall effect (Zhu et al., 2012). In contrast, more recent approaches offer an unbiased strategy for identifying genes associated with disease by comparing SNP allele frequencies across the entire genome between cases and controls. These include genome-wide association studies (GWAS) and whole-exome sequencing (WES), the latter targeting protein-coding regions of the genome (Rabbani et al., 2014).

Understanding genetic predisposition in critical illness may elucidate disease mechanisms, enable patient risk stratification, predict treatment response, and reveal novel therapeutic targets. This review presents the current understanding of genetic associations in sepsis and ARDS, explores potential mechanisms underlying critical illness, and proposes a novel strategy for studying these associations *in vitro*.

## 2 Search strategy and study grouping

**FIGURE 1:**
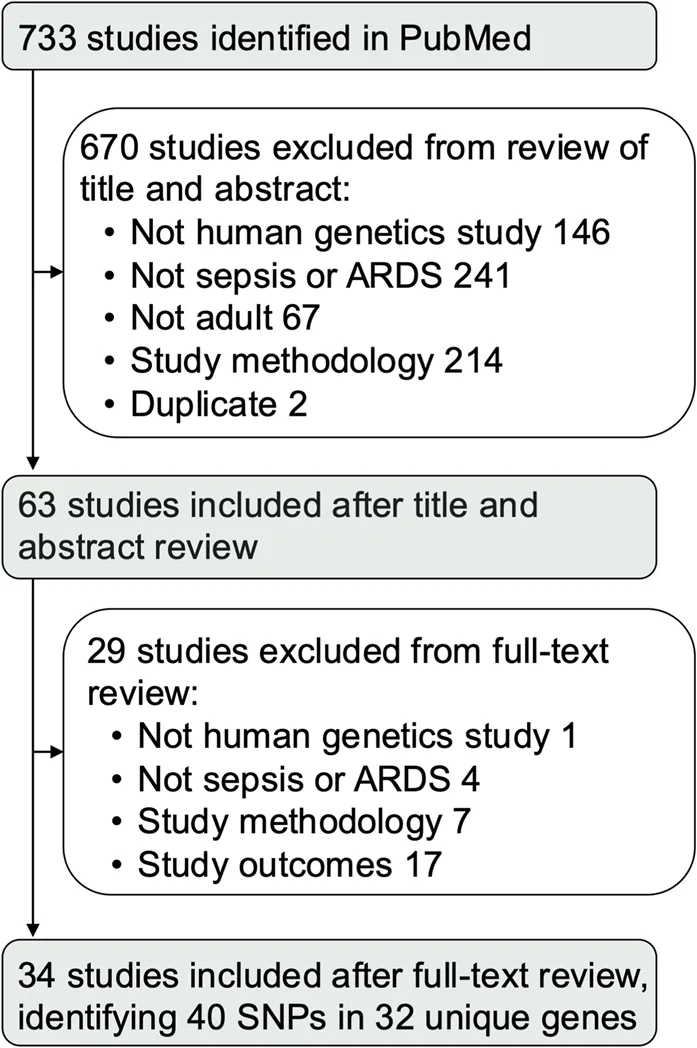
Screening and selection of studies evaluating genetic variants associated with sepsis and ARDS. A search of the literature was conducted using the PubMed database. The terms used in the search were: “sepsis”, “septic”, “acute respiratory distress syndrome”, and “ARDS”, in combination with “SNP”, “polymorphism”, “gene variant”, and “genetic variant”, as well as “GWAS”, “genome wide association study”, “genetic association study”, and “meta-analysis”. No restrictions were applied, except for publication language in English. Two investigators independently identified eligible studies for inclusion. To be included, studies had to be genome-wide studies or meta-analyses (>500 patients in each arm) of candidate gene studies. Furthermore, study participants needed to be adults and have a clinical diagnosis of sepsis or ARDS. The SNPs needed to be statistically significant for association with sepsis and ARDS in the overall study population. Animal experiments, case reports, commentaries and letters, reviews, editorials, meeting abstracts, poster presentations, and correspondence were excluded.

We searched the National Center for Biotechnology Information (NCBI) PubMed database to identify English-language manuscripts reporting genetic associations in sepsis and ARDS among critically ill adults (Figure 1). The search date was 22 September 2023. Search terms included “sepsis,” “septic,” “acute respiratory distress syndrome,” and “ARDS,” in combination with “SNP,” “polymorphism,” “gene variant,” “genetic variant,” “GWAS,” “genome-wide association study,” “genetic association study,” and “meta-analysis.” Only genome-wide studies (i.e., GWAS or WES) and meta-analyses of candidate gene studies that included more than 500 patients in each arm were considered. Eligible studies were required to report a statistically significant association between specific SNPs and sepsis and/or ARDS risk or survival. Animal experiments, case reports, commentaries, letters, reviews, editorials, meeting abstracts, poster presentations, and correspondence were excluded. Two investigators independently screened studies for inclusion.

## 3 Results

The first genetic associations in sepsis and ARDS were identified through candidate gene studies, observational cohort studies that examine predetermined SNPs in genes with a plausible mechanistic role in sepsis biology. Allele frequencies have been compared between critically ill patients and appropriate controls to determine whether a specific genotype is associated with the disease state. This approach requires minimal access to modern sequencing facilities; however, they are limited by their small sample size, poor reproducibility, and low generalizability (Sutherland and Walley, 2009).

Given the small sample sizes in many candidate gene studies, dozens of meta-analyses have been conducted to more confidently ascertain the presence of an effect (Haidich, 2010). SNP associations from candidate gene studies that remained significant in meta-analyses were included. SNPs were identified in genes related to pathogen recognition, cytokine signaling, and endothelial cell function.

Eleven GWAS and two WES studies were eligible for inclusion, identifying 19 unique SNPs in 17 distinct genomic loci associated with sepsis and/or ARDS (Table 1). In contrast to the candidate-gene meta-analyses presented in Table 2, these studies employed genome-wide approaches to identify associations without restricting analyses to pre-specified genes. SNPs were identified in genes involved in pathogen recognition, cytokine signaling, leukocyte recruitment and adhesion, and endothelial cell function, with several pathways overlapping those identified by candidate-gene studies.

**TABLE 1:** Genome-wide studies (GWAS/WES) identifying SNPs associated with sepsis and ARDS.

Gene (reference)	Chromosome	Gene function	Variant type	Variant ID	Effect allele	Functional significance of variant	Study reporting association with sepsis	Sample	Cases	Controls	EAF (%)	Outcome reported	OR (95% CI)
*Pathogen recognition*													
*CRISPLD2* (Scherag et al., 2016)	13	LPS-binding protein	Intronic	rs2641697	G	Unknown	GWAS	Sepsis	936	NA	36	Mortality	0.91 (NR)
*Cytokine signaling*													
*CISH* (Rosier et al., 2021)	3	Negative regulator of cytokine signaling	Intergenic	rs-143356980	T	Associated with reduced expression of CISH	GWAS	Septic Shock	832	NA	1	Mortality	NR
*IL1RN* (Meyer et al., 2013)	2	Competitively inhibits IL1 receptor	Exonic (synonymous)	rs315952	C	Associated with higher levels of IL1Ra protein	GWAS	ARDS vs. ICU controls	1,241	1824	17	Risk	0.81 (0.72–0.91)
*Leukocyte-endothelial cell interactions*													
*SELPLG* (Bime et al., 2018)	12	Mediates leukocyte tethering and rolling interactions with activated endothelial cells	Exonic (Met62Ile)	rs2228315	T	Hypothesized to affect PSGL-1 binding affinity to its receptor P-selectin, potentially having a functional effect on neutrophil rolling	GWAS	ARDS vs. ICU controls	372^a^	384^a^	27–31^a^	Risk	1.38 (1.10–1.72)
*SVEP1* (Nakada et al., 2015a)	9	Involved in cell adhesion	Exonic (Gln581His)	rs10817033	C	Associated with impaired SVEP1 protein function based on *in silico* modeling	Genetic association study investigating non-synonymous SNPs	Septic Shock	520	NA	11.92	Mortality	1.72 (1.31–2.26)
*Endothelial cell function*													
*FLT1* (Guillen-Guio et al., 2020; Hernandez-Pacheco et al., 2018)	13	Encoded in two forms, a transmembrane form (VEGF receptor 1), which regulates several vascular functions, and a soluble form (sFLT1), which is an endogenous inhibitor of VEGF	Intronic	rs9508032	T	Hypothesized to reduce expression of canonical VEGF receptor 1, thus decreasing VEGF signaling	GWAS	Sepsis with ARDS vs. sepsis without ARDS	633	1,302	71–75	Risk	0.61 (0.41–0.91)
			Intronic	rs9513106	C	Hypothesized to increase sFLT1 levels, protecting the lung from injury by reducing VEGF signaling and vascular permeability	GWAS	ARDS vs. healthy controls	886	1,133	22.1	Risk	0.77 (0.65–0.92)
*Other functions*													
*AL589740.1* (D'Urso et al., 2020)	6	NA^b^	Intergenic	rs9489328	T	Unknown	GWAS	Septic Shock vs. healthy controls	493	2,442	10	Risk	NR
*ARSD* (Shortt et al., 2014)	X	Lysosomal enzyme with an undiscovered function; thought to play a role in sphingolipid metabolism	Intronic	rs78142040	T	Unknown	WES	ARDS vs. healthy controls	213	625	22	Risk	498.09 (30.83–8,047.51)
*FER* (Rautanen et al., 2015)	5	Involved in cell adhesion, chemotaxis, and cytoskeletal regulation	Intronic	rs4957796	C	Unknown	GWAS	Sepsis	2078	NA	16.46	Mortality	0.56 (0.45–0.69)
*FOXO1* (Wang et al., 2019)	13	Encodes a transcription factor involved in glucose metabolism and lipogenesis	Intronic	rs2721068	T	Associated with higher serum levels of FOXO1 protein	WES	Sepsis vs. healthy controls	156	149	NR	Risk	D: 3.24 (1.25–8.44)
			Intronic	rs17446614	A	Associated with higher serum levels of FOXO1 protein	WES	Sepsis vs. healthy controls	156	147	10–20	Risk	R: 0.47 (0.026–0.88)
*LINC00378/MIR3169* (Hernand et al., 2022)	13	NA^b^	Intergenic	rs-138347802	C	Unknown	GWAS	Sepsis	2,750	NA	1.4	Mortality	2.57 (1.82–13.03)
*q21.33 locus* (Scherag et al., 2016)	13	NA^b^	Intergenic	rs9529561	G	Unknown	GWAS	Sepsis	936	NA	8	Mortality	1.87 (NR)
*SAMD9* (Hernand et al., 2022)	7	Not fully elucidated, involved in viral host defense mechanisms, cell proliferation, endosomal fusion, and tumor suppression	Exonic (Ala1556 Thr)	rs34896991	T	Unknown	GWAS	Sepsis	2,750	NA	1.8	Mortality	1.64 (1.37–6.78)
*SLC5A12/FIBIN* (Hernand et al., 2022)	11	NA^b^	Intergenic	rs-146257041	G	Unknown	GWAS	Sepsis	2,750	NA	1.5	Mortality	4.59 (2.77–7.59)
*VPS13A* (Scherag et al., 2016)	9	Encodes the protein chorein, which contributes to cytoskeletal stability and regulation	Exonic	rs-117983287	A	Unknown	GWAS	Sepsis	936	NA	1	Mortality	1.47 (NR)
*VPS13D* (Nakada et al., 2015b)	1	Encodes a ubiquitin-binding protein playing an essential role in mitophagy	Intronic	rs6685273	C	Associated with altered proportion of transcript variants of VPS13D, as well as with higher levels of IL6 *in vitro*	GWAS	Sepsis	580	NA	NR	Mortality	NR
*XKR3* (Shortt et al., 2014)	22	Encodes a homolog of XK, a putative membrane transporter	Exonic	rs9605146	A	Unknown	WES	ARDS vs. healthy controls	213	625	38.6	Risk	15.16 (10.25–22.41)

Abbreviations: A = allelic model; ARDS, acute respiratory distress syndrome; CI, confidence interval; D = dominant model; EAF, effect allele frequency; GWAS, genome-wide association study; OR, odds ratio; NA, not applicable; NR, note reported; R = recessive model; SNP, single nucleotide polymorphism; WES, whole exome sequencing. Findings in Table 1 are derived from GWAS/WES, studies and are presented separately from candidate-gene meta-analyses (Table 2).

^a^ For African American population.

^b^ Function not well characterized.

**TABLE 2:** Candidate-gene meta-analyses identifying SNPs associated with sepsis and ARDS.

Gene	Chromosome	Gene function	Variant type	Variant ID	Effect allele	Functional significance of variant	Sample	Cases	Controls	EAF (%)	Outcome reported	OR (95% CI)
*Pathogen recognition*												
*LBP* (Lu et al., 2018)	20	LPS-binding protein	Exonic (Phe436Leu)	rs2232618	T	Increased LBP affinity for CD14 on macrophages, and associated with higher TNF-α production upon stimulation with LPS *in vitro*	Sepsis vs. controls	917	1,291	81.9	Risk	D: 1.75 (1.40–2.19) R: 6.08 (1.82–20.37) A: 2.72 (2.13–3.47)
*MBL2* [(Lu et al., 2019), (Zhang et al., 2014b)]	10	MBL in plasma binds to carbohydrate structures on the surface of micro-organisms, triggering the complement system and other immune responses	Exonic	A/O haplotype (rs1800450, rs1800451, and rs5030737)	O	Associated with reduced levels of functional MBL in plasma, compromised ligand binding, and reduced complement activation	Sepsis vs. controls	3,549	5,517	12	Risk	D: 1.25 (1.05–1.48) A: 1.19 (1.04–1.36)
							Sepsis vs. controls	3,371	5,989	12	Risk	D: 1.27 (1.05–1.52) A: 1.19 (1.02–1.40)
*RAGE* (Lu et al., 2019)	6	Receptor that causes sustained activation of multiple inflammatory pathways upon activation	Intronic	rs1800625	T	Associated with increased expression of RAGE	Sepsis vs. controls	977	1,339	10–15	Risk	D: 0.59 (0.48–0.73) R: 0.47 (0.24–0.96) A: 0.61 (0.51–0.74)
*TLR1* [(Lu et al., 2019)]	4	PRR that recognizes bacterial lipoproteins and glycolipids in complex with TLR2	Intronic	rs5743551	G	Associated with elevated TLR1-mediated cytokine production	Sepsis vs. controls	1,224	943	40–45	Risk	R: 1.52 (1.16–2.00) A: 1.17 (1.02–1.34)
*TLR2* [(Gao et al., 2015)]	4	PRR that recognizes bacterial lipoproteins	Exonic (Arg753 Gln)	rs5743708	A	Blunted TLR2 response to ligands	Sepsis vs. controls	898	1,517	10.36	Risk	D: 1.92 (1.11–3.32) A: 1.76 (1.05–2.95)
*Cytokine signaling*												
*IL1B* (Lu et al., 2019; Zhang et al., 2014a; Zheng et al., 2018)	2	Inflammatory cytokine	Exonic (synonymous)	rs143634/rs1143634	C	Associated with lower levels of IL-1β protein	Sepsis vs. controls	1,095	1,168	20–30	Risk	R: 0.53 (0.34–0.82)
							Sepsis vs. healthy controls	680	998	20–30	Risk	R: 0.59 (0.36–0.97)
*IL1RN* (Zhang et al., 2014a; Fang et al., 2015)	2	Competitively inhibits IL1 receptor	VNTR	rs2234663	L	Associated with higher levels of IL1Ra protein	Severe Sepsis vs. mixed controls	1731	2,199	70.63	Risk	D: 0.67 (0.47–0.90)
					2	Associated with lower levels of IL1Ra protein	Sepsis vs. healthy controls	701	1,227	NR	Risk	A: 1.40 (1.01–1.95)
*IL6* [(Lu et al., 2019)]	7	Inflammatory cytokine	Intronic	rs1800795	C	Associated with higher levels of IL6	Sepsis vs. controls	1903	4,573	15–20	Risk	A: 1.17 (1.01–1.35)
*IL8* [(Lu et al., 2019)]	4	Inflammatory cytokine	Intronic	rs4073	T	Associated with higher levels of IL8	Sepsis vs. controls	1,314	1,605	50–60	Risk	D: 0.71 (0.60–0.84) R: 0.71 (0.52–0.97) A: 0.76 (0.63–0.92)
*IL10* [(Zhang et al., 2023)]	1	Anti-inflammatory cytokine	Intronic	rs1800896	A	Associated with higher levels of IL10	Sepsis vs. controls	3,011	2,976	40–50	Risk	R:1.32 (1.05–1.65)
*LTA* (Tiancha et al., 2011)	6	Inflammatory cytokine	Intronic	rs909253	A	Associated with altered levels of TNF‐α and TNF‐β	Sepsis	626	2,429	30	Mortality	R: 1.89 (1.27–2.80)
							Sepsis vs. controls	3,790	3,548	30	Risk	R: 1.33 (1.09–1.62)
*TNF-α* (Lu et al., 2019; Teuffel et al., 2010; Zhang et al., 2017a; Wang et al., 2017; Zhang et al., 2017b)	6	Inflammatory cytokine	Intronic	rs1800629	A	Associated with higher levels of TNF-α	Sepsis vs. controls	NR^a^	NR^a^	15–20	Risk	D: 2.15 (1.45–3.19)
							Sepsis vs. controls	NR	NR	15–20	Risk	H: 1.43 (1.07–1.92) D: 1.42 (1.06–1.89)
							Sepsis vs. controls	NR	NR	15–20	Risk	D: 1.35 (1.10–1.67)
							Septic Shock vs. controls	NR	NR	15–20	Risk	D: 1.52 (1.18–1.95)
			Intronic	rs361525	A	Associated with higher levels of TNF-α	Sepsis vs. controls	808	1,457	5–10	Risk	R: 4.88 (2.19–10.87)
*Endothelial cell function*												
*ACE* (Hou et al., 2015; Deng et al., 2015; Hu et al., 2010; Pabalan et al., 2021; Tsantes et al., 2013)	17	Converts angiotensin I to angiotensin II	Intronic	rs1799752	D	Increased ACE production	Sepsis vs. healthy controls	735	1902	26.1	Risk	R: 0.75 (0.62–0.91)
							ARDS vs. controls	NR^b^	NR^b^	NR	Risk	R: 1.57 (1.30–1.89)
							ARDS vs. controls	514	2,619	NR	Risk	R: 1.56 (1.22–2.00)
							ARDS vs. ICU controls	2,214	781	NR	Risk	R: 0.49 (0.39–0.61)
							ARDS	NR	NA	NR	Mortality	A: 1.76 (1.19–2.59)
*PAI-1* (Li et al., 2013; Shi et al., 2015)	7	Inhibits plasminogen activators	Intronic	rs1799889	4G	Associated with higher levels of PAI-1	Sepsis vs. healthy controls	1806	2,239	41.81	Risk	R: 1.30 (1.08–1.56)
							Sepsis	1806	NA	41.81	Mortality	R: 1.72 (1.27–2.33)
*Other pathways*												
*FCGR2A* (Lu et al., 2019)	1	Receptor on the surface of phagocytes that binds IgG, mediating the removal of antigen-antibody complexes from the circulation	Exonic (His131Arg)	rs1801274	G	Associated with altered immune response to infectious agents and overproduction of cytokines	Sepsis vs. controls	754	1,307	30–50	Risk	R: 1.65 (1.08–2.53) A: 1.25 (1.09–1.44)
*PCSK9* (Genga et al., 2018)	1	Inhibits the clearance of low-density lipoprotein cholesterol from circulation	Exonic (Arg46Leu)	rs11591147	A	Loss-of-function variant	Sepsis	1,481	NA	14.61	Mortality	R: 0.69 (0.50–0.94)
			Exonic (Ala53Val)	rs11583680	G	Loss-of-function variant	Sepsis	1,481	NA	15.35	Mortality	R: 0.69 (0.50–0.94)
			Exonic (Ile474Val)	rs562556	A	Loss-of-function variant	Sepsis	1,481	NA	1.02	Mortality	R: 0.69 (0.50–0.94)

Abbreviations: A = allelic model; ARDS, acute respiratory distress syndrome; CI, confidence interval; D = dominant model; EAF, effect allele frequency; GWAS, genome-wide association study; OR, odds ratio; NA, not applicable; NR, note reported; R = recessive model; SNP, single nucleotide polymorphism; WES, whole exome sequencing. Findings in Table 2 are derived from candidate-gene meta-analyses and are presented separately from genome-wide studies (Table 1).

^a^ Total sample size was 2,977.

^b^ Total sample size was 5,218.

## 4 Role of SNPs in critical illness

**FIGURE 2:**
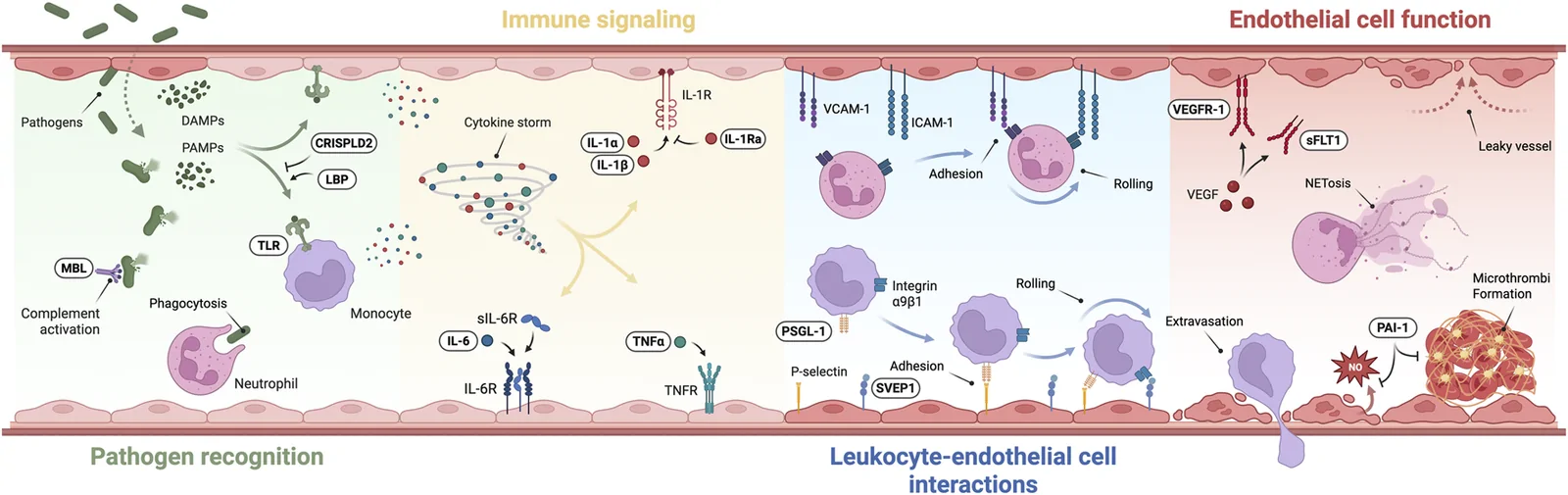
SNP-driven modulation of host immune and endothelial responses in critical illness. Figure was made with BioRender.com.

The genetic associations in sepsis and ARDS point to four key biological pathways, including pathogen recognition, immune signaling, leukocyte-endothelial cell interactions, and endothelial cell function (Figure 2). SNPs may affect these pathways by changing protein expression levels, modifying protein structure and function, and altering interactions with other genes and/or proteins. In addition, the net effect of a SNP may depend on its interactions with other SNPs and on non-inheritable factors, such as interactions with the causative organism or the anatomic source of infection. Next, we review the role of specific SNPs linked to these key biological pathways.

### 4.1 Pathogen recognition

Pattern-recognition receptors (PRRs) are central components of the innate immune system that recognize pathogen-associated molecular patterns (PAMPs) on microorganisms and danger-associated molecular patterns (DAMPs) released by the host upon tissue injury (Wiersinga et al., 2014; Delano and Ward, 2016), thereby initiating an immune response.

#### 4.1.1 TLR1 and TLR2

Toll-like receptor 1 (TLR1) and Toll-like receptor 2 (TLR2) are cell-surface receptors that bind bacterial lipoproteins, such as lipopolysaccharide (LPS) or lipoteichoic acid (Duan et al., 2022). rs5743551 and rs5743708 are SNPs located within *TLR1* and *TLR2*, respectively, and are associated with an increased risk of sepsis (Table 2). rs5743551 is located within an intron of *TLR1* and is associated with elevated TLR1-mediated cytokine production (Wurfel et al., 2008). rs5743708 is an exonic *TLR2* variant that results in a missense substitution, replacing the wild-type alanine with glycine. This SNP is associated with a reduced TLR2 response (Xiong et al., 2012) and an increased risk of gram-positive infection (Lorenz et al., 2000). In a study evaluating 91 patients with septic shock, 9% of patients with gram-positive infection were rs5743708 carriers, while the SNP was absent in patients with other sources of infection (Lorenz et al., 2000). Altering TLR1/TLR2-mediated responses may be particularly relevant in gram-positive infections, as TLR2 signaling is triggered by peptides from gram-positive organisms, with a lower response to gram-negative peptides (i.e., LPS) compared to TLR4.

#### 4.1.2 MBL2

Mannose-binding lectin (MBL) is a soluble PRR that binds to carbohydrate structures on the surface of many microorganisms (Gordon et al., 2006). MBL activates the complement system in an antibody-independent manner, modulates inflammation, and promotes phagocytosis (Gordon et al., 2006). MBL deficiency predisposes individuals to infection (Gordon et al., 2006). rs1800450, rs1800451, and rs5030737 are markers for a haplotype within the *MBL2* gene (i.e., the A/O haplotype), which denotes a grouping of genomic variations inherited together with *MBL2*. The A/O haplotype affects MBL expression and is associated with sepsis risk (Gordon et al., 2006) (Table 2). Individuals who are heterozygous for the A/O haplotype have reduced circulating MBL levels, whereas those who are homozygous (O/O) have negligible circulating MBL levels (Gordon et al., 2006). Patients with the O/O haplotype may be at increased risk of developing sepsis due to reduced ability to recognize and clear pathogens. Supporting this, studies in *MBL-*null knockout mice demonstrate that the MBL complement pathway plays a non-redundant role in defending against blood-borne *Staphylococcus aureus* infection (Shi et al., 2004).

#### 4.1.3 LBP

Lipopolysaccharide-binding protein (LBP) plays an important role in pathogen recognition by binding LPS, which is ubiquitously expressed by gram-negative bacteria (Zeng et al., 2012). LBP facilitates the interaction between LPS and macrophages by transferring LPS to membrane-bound CD14, inducing inflammatory cytokine production and immune signaling (Zeng et al., 2012). rs2232618 is a SNP located within an exon of the *LBP* gene that results in a missense mutation (F436L). The variant protein has an increased affinity for macrophage CD14 and triggers higher production of inflammatory cytokines upon LPS stimulation *in vitro* (Zeng et al., 2012). The variant is also associated with an increased risk of sepsis (Table 2).

#### 4.1.4 CRISPLD2

Cysteine-rich secretory protein LCCL domain containing 2 (CRISPLD2) is an LPS-binding protein that blunts the immune response by sequestering LPS (Wang et al., 2009). Decreased expression of CRISPLD2 is associated with septic shock and inversely correlated with procalcitonin levels (Wang et al., 2013). rs2641697 is an intronic *CRISPLD2* variant associated with sepsis mortality and may influence protein expression. (Scherag et al., 2016). Recombinant CRISPLD2 (rCRISPLD2) administration in mice demonstrates a protective and anti-inflammatory effect. For example, injection of *Escherichia coli* LPS in combination with rCRISPLD2 reduced animal mortality from 80% to 20%, suggesting that CRISPLD2 may have a protective anti-endotoxin effect (Wang et al., 2009). rCRISPLD2 administration also reduces serum levels of inflammatory cytokines in septic mice (Zhang et al., 2021).

### 4.2 Immune signaling

#### 4.2.1 Pro-inflammatory cytokines

Following PRR activation, the transcription factor NF‐κβ triggers an inflammatory cascade that results in cytokine release (Bode et al., 2023), with tumor necrosis factor alpha (TNF-α), interleukin-6 (IL-6), and interleukin-8 (IL-8) as key upregulated cytokines (Abraham and Kroeger, 1999; Lomeli-Nieto et al., 2019; Panoulas et al., 2009; Stoica et al., 2010; Hull et al., 2000). rs1800629/rs361525, rs1800795, and rs4073 are SNPs located within introns of *TNF-α*, *IL-6*, and *IL-8*, respectively (Abraham and Kroeger, 1999; Lomeli-Nieto et al., 2019; Panoulas et al., 2009; Stoica et al., 2010; Hull et al., 2000) (Table 2). These SNPs are associated with increased cytokine production compared to individuals who do not carry the SNPs (Abraham and Kroeger, 1999; Lomeli-Nieto et al., 2019; Panoulas et al., 2009; Stoica et al., 2010; Hull et al., 2000). Targeting one or more of these pro-inflammatory cytokines with blocking monoclonal antibodies may confer a survival benefit in patients with critical illness. The MONARCS randomized clinical trial evaluated the use of an anti-TNF-α monoclonal antibody (Afelimomab) in 2,634 patients with sepsis, demonstrating a nearly 12% relative risk reduction in death among patients with serum IL-6 levels >1,000 pg/mL (Panacek et al., 2004). In addition, a Mendelian randomization (MR) analysis found that functional downregulation of IL-6 signaling pathways may improve outcomes in sepsis (Hamilton et al., 2023). An MR analysis is an observational study that uses genetic variation to examine the causal relationships between an exposure (IL-6 level) and an outcome (mortality). In an MR analysis, the exposure is linked to a genetic variant (a SNP), serving as a proxy for the exposure. In the study above, IL-6 blockade, as proxied by SNPs that lower IL-6 activity, was causally associated with lower sepsis incidence (Hamilton et al., 2023).

The variable efficacy of cytokine-targeted therapies observed in sepsis trials may partially reflect underlying genetic heterogeneity among patients. SNPs within inflammatory genes such as *TNF-α*, *IL-6*, and *IL-8* can influence cytokine production and downstream signaling, potentially affecting both disease severity and responsiveness to immunomodulatory therapies. Future pharmacogenomic studies may help identify genetically defined subgroups that derive greater benefit from targeted cytokine inhibition, thereby enabling more personalized therapeutic approaches in critical illness.

#### 4.2.2 CISH

Cytokine inducible SH2 (CISH) is a negative regulator of cytokine signaling. rs143356980 is a SNP located in the enhancer region of the *CISH* gene that is associated with reduced CISH expression and an increased risk of sepsis (Rosier et al., 2021).

#### 4.2.3 The IL-1 pathway

Several genetic association studies have implicated the IL-1 signaling pathway in critical illness syndromes (Lu et al., 2019; Zhang et al., 2014a; Fang et al., 2015; Meyer et al., 2013; Meyer et al., 2014). Interleukin-1 beta (IL-1β) is a pro-inflammatory cytokine that plays a major role in the innate immune response (Lu et al., 2019; Zhang et al., 2014a). rs143634 is a SNP within the *IL-1β* gene associated with higher circulating IL-1β (Lu et al., 2019; Zhang et al., 2014a).

IL-1 receptor antagonist (IL1Ra), encoded by the *IL1RN* gene, is a member of the IL-1 family that binds to IL-1 receptors but does not induce intracellular responses, thereby acting as a negative regulator of the IL-1 pathway (Meyer et al., 2013; Meyer et al., 2014). rs315952 is a SNP located within the *IL1RN* gene that confers a protective effect in sepsis-associated ARDS (Meyer et al., 2013) (Table 1) and is associated with improved survival in septic shock (Meyer et al., 2014). Functional analyses demonstrated that this SNP is associated with higher plasma levels of the IL1Ra protein (Meyer et al., 2013). Interestingly, another variant in the *IL1RN* gene, rs2234663, is associated with lower IL1Ra levels and an increased risk of sepsis (Table 2).

The administration of recombinant IL1Ra (rIL1Ra; Anakinra) may have a protective effect in sepsis and ARDS. Unfortunately, Anakinra failed to reduce sepsis mortality (Fisher et al., 1994; Opal et al., 1997). A double-blind, placebo-controlled, multicenter study of 893 patients with sepsis did not demonstrate a survival advantage for those receiving rIL1Ra compared with placebo (Fisher et al., 1994). Relative to placebo, rIL1Ra dosing of 1 and 2 mg/kg per hour reduced 28-day mortality by 9% and 15%, respectively, without reaching statistical significance (Fisher et al., 1994). Another double-blind, placebo-controlled, multicenter trial including 906 patients found no significant difference in 28-day mortality between patients who received rIL1Ra (18.3%) and those who received placebo (24.2%) (Opal et al., 1997). However, a post hoc analysis of these trials revealed that the response to Anakinra may be influenced by plasma IL1Ra concentration (Meyer et al., 2018), as Anakinra significantly reduced mortality in patients with baseline plasma IL1Ra levels >2,071 pg/mL (Meyer et al., 2018). These findings suggest that the administration of rIL1Ra to septic patients with high plasma IL1Ra, which is partly driven by SNPs (Meyer et al., 2013), may improve survival. More broadly, they highlight the potential role of pharmacogenomics in critical illness, whereby genetic variation within *IL1RN* may contribute to differences in endogenous IL1Ra production, consequently resulting in differential treatment effects. Future precision clinical trials integrating both genetic and biomarker-based patient stratification may help identify individuals most likely to benefit from Anakinra and other targeted immunomodulatory therapies.

### 4.3 Leukocyte-endothelial cell interactions

Inflammation exposes adhesion molecules expressed on endothelial cells, including intracellular adhesion molecule‐1 (ICAM‐1) and vascular adhesion molecule‐1 (VCAM‐1) (Dolmatova et al., 2021). This process enables leukocyte rolling, adhesion, and transmigration into tissues (Dolmatova et al., 2021).

#### 4.3.1 The selectin family

Genetic association studies have implicated genes within the selectin family of adhesion molecules. Selectin P ligand (*SELPLG*) codes for P-selectin glycoprotein ligand-1 (PSGL-1), which mediates leukocyte tethering and rolling interactions with activated endothelial cells (Bime et al., 2018). PSGL-1 binds to P-selectin on endothelial cells to facilitate these interactions (Bime et al., 2018). rs2228315 is a coding SNP that results in a missense mutation in PSGL-1 (M62I). The functional significance of the rs2228315 SNP is not known; however, it could affect the binding affinity of PSGL-1 to P-selectin, which could influence neutrophil rolling (Bime et al., 2018). PSGL-1 may be a novel therapeutic target in ARDS as mice pretreated with PSGL-1-neutralizing antibody exhibited reduced lung inflammation and injury in response to LPS (Bime et al., 2018). Specifically, the co-administration of PSGL-1 antibody and LPS resulted in a significant decrease in LPS-induced bronchoalveolar lavage total protein and cell counts. Furthermore, alveolar wall thickness and lung injury scores were reduced in mice treated with the PSGL-1 antibody (Bime et al., 2018). When compared with wild-type C57/B6 mice, *SELPLG* knock-out mice demonstrated similar attenuation of LPS-induced lung inflammation and injury (Bime et al., 2018). These results suggest that increased PSGL-1 activity and SELPLG expression may increase susceptibility to ARDS. Although experimental models support a role for PSGL-1 in ARDS-related inflammation and lung injury, the direct functional effects of the rs2228315 variant on PSGL-1 activity and leukocyte trafficking during sepsis and ARDS remain to be fully elucidated.

#### 4.3.2 SVEP1

SVEP1 is an adhesion protein related to the selectin family, is a ligand for integrin α9β1, and mediates cell adhesion (Nakada et al., 2015a). rs10817033 is a coding SNP within the *SVEP1* gene, resulting in a missense mutation in the resultant protein (Q581H). This SNP is associated with increased mortality based on WES in a cohort of 520 patients with septic shock (Table 1) (Nakada et al., 2015a). While the consequence of this mutation has not been functionally validated, *in silico* modeling predicts that it could impair SVEP1 protein function by altering the protein’s structure, as the mutation is located within a highly conserved region of the protein sequence (Nakada et al., 2015a). While these findings support a potential role for SVEP1 in sepsis susceptibility and outcomes, the functional consequences of the Q581H variant and its mechanistic contribution to sepsis pathophysiology remain unknown.

### 4.4 Endothelial cell function

The endothelium plays a crucial role in inflammation and vascular barrier integrity (Arina and Singer, 2021; Dolmatova et al., 2021). In sepsis, ARDS, and other critical illness syndromes, a complex inflammatory cascade is activated, culminating in capillary barrier dysfunction, increased vascular permeability, interstitial tissue edema, and, ultimately, end-organ damage (Arina and Singer, 2021; Dolmatova et al., 2021).

#### 4.4.1 FLT1

Fms-related tyrosine kinase 1 (*FLT1*) encodes the vascular endothelial growth factor receptor 1 (VEGFR-1), which plays an important role in vascular function (Shibuya, 2013). VEGFR-1 is encoded in two forms: a transmembrane form and a soluble form (sFLT1). The binding of VEGF to the transmembrane form of VEGFR-1 contributes to VEGF-mediated signaling, inducing vascular permeability. In contrast, sFLT1 acts as an endogenous inhibitor of the VEGF pathway by sequestering VEGF from its receptors (Shibuya, 2013). rs9508032 is a SNP located in intron 10 of *FLT1*, and is associated with reduced *FLT1* expression. rs9508032 is associated with reduced susceptibility to sepsis-associated ARDS (Table 1) (Guillen-Guio et al., 2020). While the functional significance of rs9508032 is unknown, preliminary work suggests that it may reduce the expression of the transmembrane form of VEGFR-1, resulting in decreased VEGF signaling (Hernandez-Pacheco et al., 2018). Another SNP in *FLT1*, rs9513106, also reduces the risk of sepsis-associated ARDS, potentially by increasing sFLT1 levels and leading to the downregulation of VEGF signaling (Hernandez-Pacheco et al., 2018). These SNPs may reduce VEGF-mediated vascular permeability and endothelial damage observed in sepsis and ARDS. Targeting the VEGF pathway in patients with relevant polymorphisms may identify patients likely to benefit from anti-VEGF therapy.

#### 4.4.2 ACE

Angiotensin Converting Enzyme (ACE) is a key component of the renin-angiotensin system (RAS), catalyzing the conversion of angiotensin I to angiotensin II, a potent vasoactive peptide involved in the regulation of vascular tone and systemic blood pressure (Rigat et al., 1990). The *ACE* insertion/deletion (I/D) polymorphism (rs1799752), characterized by the presence or absence of a 287 base pair sequence in intron 16, has been associated with altered serum ACE activity, with double deletion (DD) carriers demonstrating substantially higher ACE levels compared to non-deletion carriers (Rigat et al., 1990). Five meta-analyses have demonstrated an association between the *ACE* I/D polymorphism and sepsis/ARDS susceptibility, with the DD genotype associated with reduced risk of sepsis and ARDS (Table 2) (Hou et al., 2015; Deng et al., 2015; Hu et al., 2010; Pabalan et al., 2021; Tsantes et al., 2013). Given the importance of RAS in regulating vascular tone during shock, increased ACE activity may confer physiologic benefit in sepsis by improved blood pressure regulation. However, functional studies are required to clarify the mechanistic relationship between *ACE* polymorphism, sepsis susceptibility, and hemodynamic regulation.

#### 4.4.3 PAI-1

Plasminogen activator inhibitor 1 (PAI-1) plays a key role in the coagulation cascade by binding plasminogen activators and inhibiting fibrinolysis. rs1799889 is an intronic SNP within *PAI-1* associated with altered expression of PAI-1 (Table 2). The variant that leads to elevated PAI-1 levels is associated with an increased risk of sepsis and higher mortality (Li et al., 2013; Eriksson et al., 1995). PAI-1 may contribute to endothelial dysfunction by inhibiting endothelial nitric oxide synthase (eNOS), facilitating neutrophil recruitment, promoting vascular inflammation, and inducing tissue injury (Badran and Gozal, 2022). As such, higher PAI-1 levels might increase the risk of sepsis and contribute to poorer outcomes.

### 4.5 Other pathways

#### 4.5.1 VPS13A and VPS13D

*VPS13A* encodes a member of the vacuolar protein sorting (VPS13) family involved in intracellular protein trafficking and autophagic degradation pathways (Scherag et al., 2016). A potentially deleterious missense variant within *VPS13A* (rs117983287) demonstrated a strong association signal for 28-day sepsis mortality in a GWAS (Scherag et al., 2016). Experimental studies have suggested that VPS13A plays an important regulatory role in autophagy, the cellular process that mediates the degradation of cellular components in lysosomes, which is critical for immune homeostasis, pathogen clearance, and regulation of inflammatory signaling (Scherag et al., 2016). Impaired autophagy has been associated with mitochondrial dysfunction, exaggerated inflammatory responses, and organ injury in critical illness (Cuervo and Macian, 2014; Schneider and Cuervo, 2014). Given the emerging role of autophagy and mitophagy in sepsis pathophysiology, VPS13A-mediated alterations in cellular degradation pathways may influence host immune responses and clinical outcomes in patients with sepsis. Additional functional studies are required to clarify the precise mechanistic relationship between *VPS13A* variants and sepsis-related inflammation.

VPS13D is a member of the VPS13 family involved in intracellular protein trafficking, endosomal-lysosomal pathways, and mitochondrial maintenance (Nakada et al., 2015b). The *VPS13D* rs6685273 C allele has been associated with increased organ dysfunction and higher 28-day mortality in septic shock patients (Nakada et al., 2015b). Functional studies demonstrated that silencing *VPS13D* significantly increased IL-6 production under both basal and TNF-stimulated conditions, suggesting that VPS13D may act as a negative regulator of inflammatory cytokine signaling (Nakada et al., 2015b). Elevated IL-6 levels are strongly associated with sepsis severity and mortality (Abraham et al., 1997; Yende et al., 2008), supporting a potential mechanistic link between VPS13D dysfunction and adverse clinical outcomes. In addition, VPS13D has been implicated in mitochondrial quality-control pathways, including mitophagy, which is essential for the removal of damaged mitochondria and regulation of inflammatory responses during sepsis (Nakada et al., 2015b). Disruption of these pathways may contribute to persistent inflammation, immune dysregulation, and multiorgan dysfunction in critical illness. Collectively, these findings suggest that VPS13D may influence sepsis outcomes through combined effects on cytokine regulation, intracellular trafficking, and mitochondrial homeostasis.

#### 4.5.2 PCSK9

Protein convertase subtilisin/kexin 9 (PCSK9) is a regulator of low-density lipoprotein receptor (LDL-R) expression. It plays an important role in lipid metabolism and host inflammatory responses during sepsis (Genga et al., 2018). Loss-of-function (LOF) variants in *PCSK9* are associated with increased hepatic LDL-R expression and enhanced clearance of circulating low-density lipoprotein cholesterol (LDL-C) (Genga et al., 2018). Because pathogen-associated lipids, including endotoxins, are incorporated into circulating lipoproteins and cleared through LDL-R-mediated hepatic pathways, *PCSK9* LOF variants may also promote enhanced clearance of bacterial lipids during sepsis. In fact, *PCSK9* LOF variants are associated with reduced systemic inflammatory responses and decreased short-term mortality in septic patients (Walley et al., 2014), improved bacterial clearance in experimental models of sepsis (Dwivedi et al., 2016), and lower rates of infection-related readmission among sepsis survivors (Genga et al., 2018). Common LOF variants, including R46L (rs11591147), A53V (rs11583680), and I474V (rs562556), have been associated with reduced LDL-C levels and improved clinical outcomes in sepsis (Genga et al., 2018). Experimental animal models further support a mechanistic role for PCSK9 in infection resolution, as *PCSK9* knockout mice subjected to cecal ligation and puncture demonstrated reduced bacterial burden compared with wild-type controls (Walley et al., 2014; Dwivedi et al., 2016). Collectively, these findings suggest that reduced PCSK9 activity may confer protection during and after sepsis through enhanced endotoxin clearance and modulation of the host inflammatory response.

## 5 Translational biology

This review has identified over 30 genes belonging to several key molecular pathways that represent potential novel druggable targets for sepsis and/or ARDS. Compounds targeting the pathways related to these genes have shown efficacy in selected clinical sub-analyses (Panacek et al., 2004; Hamilton et al., 2023; Meyer et al., 2018) and in preclinical studies (Wang et al., 2009; Zhang et al., 2021; Bime et al., 2018). The molecular mechanisms by which these genes influence outcomes in critical illness remain unknown, and it is unclear which patients may respond to targeted treatment.

Historically, animal models and cell culture systems have provided preclinical safety data for new drugs, while randomized clinical trials (RCTs) have been used to determine safety and efficacy in large populations (Chandy et al., 2022). Current animal models in sepsis include mouse endotoxin (LPS) challenge, cecal ligation and puncture, and fecal slurry models (Marshall and Leligdowicz, 2022; Siempos et al., 2014; Lewis et al., 2016). These models are limited in capturing the complexities of human sepsis (Lewis et al., 2016). Limitations include considerable model variation due to age, sex, strain of mice used, magnitude of the septic insult, timing of delivery, anesthetic and antibiotic choices, and the volume and timing of fluid resuscitation (Lewis et al., 2016). There are also notable genetic differences related to the rodent genome (Lewis et al., 2016). Cell culture systems have been used to study the effects of SNPs on cell phenotype to determine how SNPs may contribute to critical illness biology (Rosier et al., 2021; Meyer et al., 2013; Guillen-Guio et al., 2020). While such an approach is useful for studying a particular SNP, it does not readily enable investigation of multiple SNP interactions or of interactions between different cell types carrying specific SNPs. Thus, novel methods are needed to characterize the genetic underpinnings of sepsis and ARDS, and to identify patients who are most likely to benefit from targeted therapies.

Induced pluripotent stem cells (iPSCs) are powerful tools for modeling complex diseases (Aboul-Soud et al., 2021), and represent a novel method for studying how SNPs associated with sepsis and/or ARDS influence the biology of critical illness. iPSCs are lab-generated stem cells reprogrammed from adult somatic cells, such as peripheral blood mononuclear cells or fibroblasts. iPSC-derived cells retain the patient’s unique genetic profile, thus enabling personalized disease modeling and drug screening (Aboul-Soud et al., 2021; Paik et al., 2020). For example, iPSCs have been generated from patients with cardiac arrhythmias (Moretti et al., 2010), cardiomyopathies (Carvajal-Vergara et al., 2010), Alzheimer’s disease (Yagi et al., 2011), Parkinson’s disease (Imaizumi et al., 2012), and other developmental (Ogawa et al., 2015) and infectious diseases (Garcez et al., 2016) to understand how genetic polymorphisms contribute to these diseases.

**FIGURE 3:**
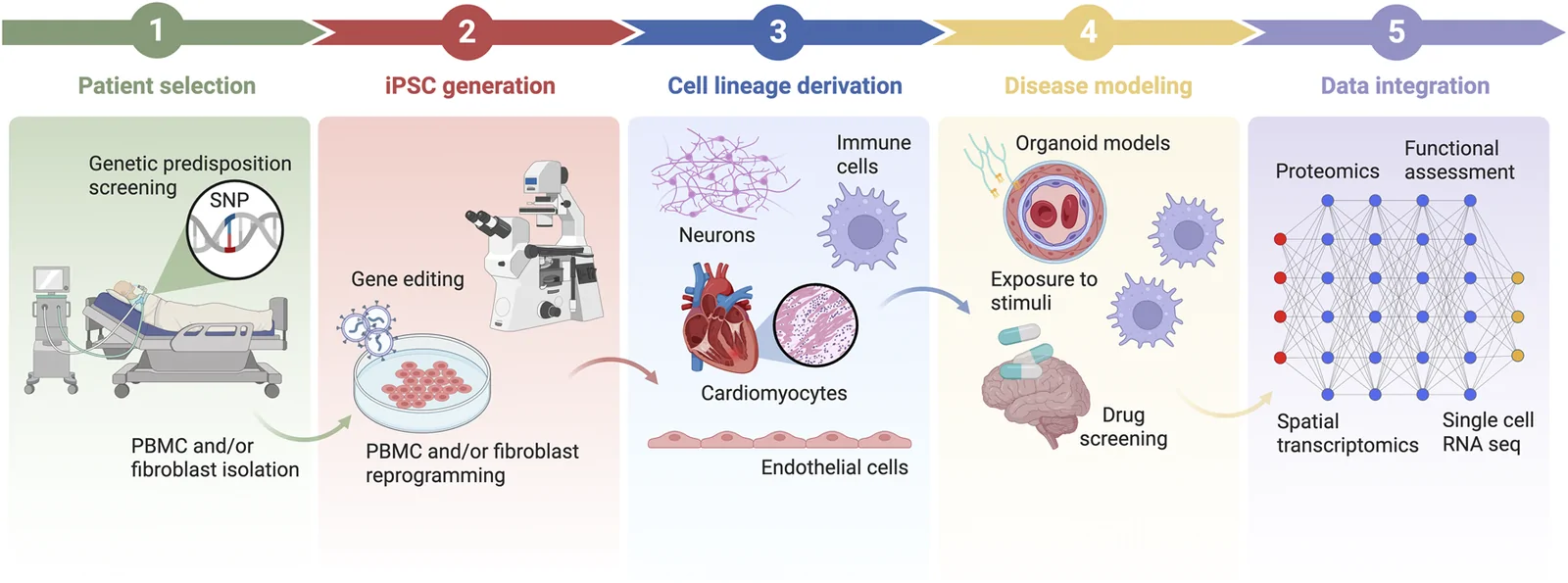
A translational pipeline for modeling SNP-associated susceptibility and pathways in critical illness. Figure was made with BioRender.com.

iPSCs could similarly be applied to critical illness (Figure 3). Once generated, iPSCs can be differentiated into almost any cell type. iPSCs derived from critically ill patients with specific genetic polymorphisms, or genetically engineered using CRISPR/Cas9-mediated introduction of candidate variants (Bassett, 2017; Singh et al., 2024), could be differentiated into endothelial cells, macrophages, and other cells implicated in the biology of critical illness. Following *in vitro* exposure to inflammatory mediators or potential pharmacologic targets that interact with pathways relevant to the gene’s function, iPSC-derived disease models could be used to investigate how sepsis- and ARDS-associated genetic variants influence inflammatory mediator production, endothelial permeability, leukocyte adhesion, mitochondrial function, and/or autophagy/mitophagy (Calvert and Ryan Firth, 2020; Cerneckis et al., 2024). Such approaches may provide mechanistic insight into how genetic variation contributes to immune dysregulation and organ dysfunction during critical illness and help establish causal links between genetic variants and clinical outcomes.

Despite their considerable promise, iPSC-based models have limitations that should be acknowledged. iPSC-derived cells can exhibit immature phenotypes that more closely resemble developmental-stage tissues rather than fully differentiated adult cells, which may limit their ability to accurately model the biology of sepsis and ARDS in adults (Cerneckis et al., 2024; Doss and Sachinidis, 2019; Volpato and Webber, 2020). Furthermore, conventional iPSC culture systems do not fully recapitulate the complex multicellular microenvironment present *in vivo*, where interactions among endothelial cells, epithelial cells, immune cells, circulating mediators, and mechanical forces collectively influence disease progression (Calvert and Ryan Firth, 2020; Cerneckis et al., 2024). Additional challenges include variability in differentiation efficiency, heterogeneity between iPSC lines, and difficulties reproducing the systemic physiologic responses that characterize critical illness (Volpato and Webber, 2020). Three-dimensional (3D) culture systems and organoids may help overcome some of these limitations by more accurately emulating tissue and organ structure.

## 6 Genetic associations in other critical illness syndromes

While this review focused on SNPs associated with sepsis and ARDS, it is important to note that GWAS studies in other critical illness syndromes have also identified polygenetic associations (Ellinghaus et al., 2020; Pairo-Castineira et al., 2023; Initiative et al., 2023). For example, a GWAS of 24,202 patients with critical COVID-19 identified 49 genetic associations related to multiple biological pathways (Pairo-Castineira et al., 2023). Notably, the study identified possible drug targets, including JAK1 (inflammatory signaling), PDE4A (monocyte–macrophage activation and endothelial permeability), SLC2A5 and AK5 (immunometabolism), and TMPRSS2 and RAB2A (host factors required for viral entry and replication) (Pairo-Castineira et al., 2023). Another study involving 1,980 COVID-19 patients with severe respiratory failure identified a 3p21.31 gene cluster (SLC6A20, LZTFL1, CCR9, FYCO1, CXCR6, and XCR1) associated with disease susceptibility (Ellinghaus et al., 2020). Since these studies identified associations in genes that were not previously implicated in sepsis or ARDS, they reaffirm the biological complexity of critical illness.

## 7 Limitations

The identification of SNPs in sepsis and ARDS has provided insight into the molecular pathways involved in critical illness. Nevertheless, as critical illness outcomes hinge on a combination of factors, including the interaction of genetic and non-genetic drivers of disease, SNPs alone cannot capture the full complexity of critical illness. Advances in multiomic data analysis may help fill these gaps by integrating complementary data to enhance our understanding of critical illness. These technologies affirm the extensive heterogeneity in critical illness (Maslove et al., 2022) and yield new insights into the pathophysiology of sepsis (Mu et al., 2023; Pelaia et al., 2023; Zhang et al., 2024; Chen et al., 2024), ARDS (Du et al., 2021; Hernandez-Beeftink et al., 2019), and other critical illness syndromes (Li et al., 2023). In parallel, emerging analytical approaches such as expression quantitative trait locus (eQTL) analysis and MR may help bridge the gap between genetic association and biological mechanism. eQTL analyses can aid in determining whether disease-associated variants influence gene expression in relevant tissues or immune cell populations, thereby prioritizing biologically plausible candidate genes within associated loci (Jacobs et al., 2020; Porcu et al., 2019). Similarly, MR approaches can provide insight into potential causal relationships between genetically regulated biomarkers, inflammatory pathways, or gene expression profiles and clinical outcomes in critical illness (Porcu et al., 2019; Zuber et al., 2022). Integration of GWAS findings with multiomic, eQTL, and MR data may therefore improve the identification of functionally relevant variants, facilitate prioritization of therapeutic targets, and help move beyond purely statistical associations toward a more mechanistic understanding of disease biology (Mountjoy et al., 2021; Sadler et al., 2023).

## 8 Conclusion

Critical illness syndromes are complex, heterogeneous, life-threatening polygenic conditions. Each genetic variant likely has an incremental effect on the development and progression of these syndromes. How these risk variants mediate the cellular mechanisms that trigger disease onset and progression remains poorly understood. Understanding the molecular mechanisms of how genotype influences biological outcomes may lead to the discovery of new druggable targets. iPSCs could enable the investigation of SNP interactions, uncover new insights into disease pathophysiology, and predict patient-specific treatment responses, providing a precision-medicine guided approach to improve outcomes in critical illness.
